# Integrated Transcriptome and Pathway Analyses Revealed Multiple Activated Pathways in Breast Cancer

**DOI:** 10.3389/fonc.2019.00910

**Published:** 2019-09-18

**Authors:** Radhakrishnan Vishnubalaji, Varun Sasidharan Nair, Khalid Ouararhni, Eyad Elkord, Nehad M. Alajez

**Affiliations:** Cancer Research Center, Qatar Biomedical Research Institute, Hamad Bin Khalifa University, Qatar Foundation, Doha, Qatar

**Keywords:** breast cancer, RNA-Seq, pathway analysis, transcriptome, IPA

## Abstract

Breast cancer (BC) is the leading cause of cancer-related death in women. Therefore, a better understanding of BC biology and signaling pathways might lead to the development of novel biomarkers and targeted therapies. Although a number of transcriptomic studies have been performed on breast cancer patients from various geographic regions, there are almost no such comprehensive studies performed on breast cancer from patients in the gulf region. This study aimed to provide a better understanding of the altered molecular networks in BC from the gulf region. Herein, we compared the transcriptome of BC to adjacent normal tissue from six BC patients and identified 1,108 upregulated and 518 downregulated transcripts. A selected number of genes from the RNA-Seq analysis were subsequently validated using qRT-PCR. Differentially expressed (2.0-fold change, adj. *p* < 0.05) transcripts were subjected to ingenuity pathway analysis, which revealed a myriad of affected signaling pathways and functional categories. Activation of ERBB2, FOXM1, ESR1, and IGFBP2 mechanistic networks was most prominent in BC tissue. Additionally, BC tissue exhibited marked enrichment in genes promoting cellular proliferation, migration, survival, and DNA replication and repair. The presence of genes indicative of immune cell infiltration and activation was also observed in BC tissue. We observed high concordance [43.5% (upregulated) and 62.1% (downregulated)] between differentially expressed genes in our study group and those reported for the TCGA BC cohort. Our data provide novel insight on BC biology and suggest common altered molecular networks in BC in this geographic region. Our data suggest future development of therapeutic interventions targeting those common signaling pathways.

## Introduction

Breast cancer (BC) is the second most common type of cancer around the world comprising approximately 11.6% of new cancer cases and 6.6% of all cancer-related deaths up to 2018 (1). Among females, BC is the most frequently diagnosed and the leading cause of cancer mortality. GLOBOCAN 2018 reported region-specific incidence and age-standardized mortality rate for BC in Western Asia (incidence: 45.3/100,000, mortality: 13.6/100,000) and Eastern Asia (incidence: 39.2/100,000, mortality: 8.6/100,000) (1).

Gene expression profiling by DNA microarray have identified the inherent classification of BC into five main molecular subtypes: Luminal A (estrogen receptor (ER) +/progesterone receptors (PR) +/epidermal growth factor receptor 2 (HER2; ERBB2) –) are commonly of low score; luminal B (ER+/PR–/+/HER2+/–) are normally of higher score with more proliferation rate; HER2-enriched subtype (ER–/PR–/HER2+); triple-negative breast cancer (TNBC; ER–/PR–/HER2–); and carcinomas that are analogous to normal breast tissue and is associated with good prognosis (2–4). In addition, claudin-low cancers, metaplastic, molecular apocrine, and invasive lobular carcinomas were identified as molecularly different BCs (5). Furthermore, genome-wide association studies have identified many novel breast cancer vulnerability variants such as hereditary risk factors, encompassing four sporadic high-penetrance transcriptomes (BRCA1, BRCA2, TP53, and PTEN), four sporadic moderate: penetrance transcriptomes (CHEK2, ATM, BRIP1, and PALB2), and around twenty common low-penetrance variants in 19 genes or loci (6, 7).

The age-standardized incidence rate is growing in many countries, particularly in the Arab countries where the reported BC incidence ranges from 9.5 to 50 cases per 100,000 women per year. In the gulf region, the incidence of BC in Bahrain, United Arab Emirates, Saudi Arabia, Qatar, and Kuwait were 53.4, 22.8, 17.5, 48.2, and 46.6 cases per 100,000 women, respectively (8). Although the incidence of BC in this geographic region is lower than those reported in Europe and USA, the incidence of BC in Arab countries are on the rise (1, 9). Interestingly, patients diagnosed with BC in the Arab world are approximately a decade younger and they are oftentimes presented with larger and more advanced stage tumors (8). A previous microarray-based study comparing the clinical and gene expression profile of breast cancer from the north (France) and south (Lebanon, Tunisia, and Morocco) Mediterranean patients revealed more aggressive tumor in the south Mediterranean patient group. Tumors from the south group were predominantly luminal B, while tumors from the north were mostly luminal A subtype (10).

Recent advances in transcriptome analysis have revolutionized our understanding of human disease (11). In the current study, we utilized next generation sequencing (NGS) and bioinformatics and characterized the transcriptional landscape of BC compared to adjacent normal tissue from the gulf region and identified multiple activated networks. Our data provides the first transcriptome and network analyses of BC in this geographic region setting the foundation for future development of novel BC biomarkers and therapeutic interventions.

## Materials and Methods

### Ethics Statement and Sample Collection

Tumor tissues (TT) and adjacent non-cancerous normal tissues (NT) were obtained from six breast cancer patients. All patients included in the study were treatment-naive prior to surgery and were provided with a written informed consent prior to sample collection. The study was performed under ethical approval from Qatar Biomedical Research Institute, Doha, Qatar (Protocol no. 2017-006). The characteristics of patients included in current study are provided in Table 1.

**Table 1:** Clinical information of patients included in current study and their tumor characteristics.

**Patient ID**	**Age**	**Ki-67**	**ER**	**PR**	**Her2**	**Histological grade**	**Type**	**TNM stage**	**Staging**
PBC-004	41	70%	Negative	Negative	Positive	Poorly differentiated	IDC	pT4b N2 Mx	IIIB
PBC-005	33	30%	Positive	Positive	Negative	NA	IDC	PT2 N0 Mx	IIA
PBC-020	59	10%	Positive	Positive	Positive	Poorly differentiated	IDC	pT2 N1 M0	IIB
PBC-041	59	20%	Positive	Positive	Positive	Moderately differentiated	IDC	pT3 N3 M0	IIIC
PBC-045	57	15%	Positive	Positive	Negative	Poorly differentiated	IDC	pT1 cN0	IA
PBC-055	43	10%	Positive	Positive	NA	Well differentiated	IDC	pT1 N0 Mx	IA

### Tissue Preparation and RNA Isolation

RNA was isolated using the RNA/DNA/Protein Purification Plus Kit (Norgen Biotek Corp, Ontario, Canada) as per the manufacturer's instructions from TT and adjacent NT. Briefly, frozen tissues were transferred into a mortar containing adequate amount of liquid nitrogen and were grinded thoroughly using a pestle followed by resuspending the tissue in lysis buffer followed by RNA extraction. The concentration and purity of extracted RNA were measured using NanoDrop 2000c (Thermo scientific, MA, USA) and RNA were stored at −80°C.

### RNA Concentration and Quality Assessment

The quality and quantity of extracted RNA was measured using on-chip electrophoresis utilizing the Agilent RNA 6000 Nano Kit (Agilent Technologies, CA, USA) and Agilent 2100 Bioanalyzer (Agilent Technologies) as per the manufacturer's instructions. Samples exhibited an RNA Integrity Number (RIN) > 7 were used for library preparation.

### Library Preparation

The RNA was quantified using Qubit instrument (Invitrogen, USA) and RNA BR assay kit (Invitrogen). Hundred nanogram of RNA was used as an input for library preparation using TruSeq RNA Access Library preparation kit (Illumina, CA, USA) as per the manufacturer's instructions. Briefly, the RNA was fragmented into small pieces under high temperature using divalent cations. The RNA fragments were immediately reverse transcribed to first strand cDNA using random hexamers. Following the first strand, second strand was synthesized by incorporating dUTP instead of dTTP. The sequencing adaptors were ligated to the double-stranded cDNA followed by a single “A” nucleotide adenylation at 3′ end of blunt fragments. The final library was created by capturing the coding regions of the transcriptome using sequence-specific probes. The yield of cDNA libraries was quantified using Qubit dsDNA HS assay kit (Invitrogen) and size distribution of the cDNA libraries were determined using the Agilent 2100 Bioanalyzer DNA1000 chip (Agilent Technologies). The clusters were generated on a cBot cluster generation system (Illumina) and sequencing was done on Hiseq 4000 with 300 bp paired-ends.

### Quantitative Reverse Transcription PCR (RT-qPCR)

One microgram of RNA from each sample was reverse transcribed into cDNA using QuantiTect Reverse Transcription Kit (Qiagen, Hilden, Germany). PCR reactions were performed on QuantStudio 7/6 Flex qPCR (Applied Biosystems, California, USA) using PowerUP SYBR Green Master Mix (Applied Biosystems). All data were normalized to β-actin. Non-specific amplifications were checked by the use of melting curve. The relative changes in target gene expression were analyzed using 2-ΔΔCT method. Sequences of primers used in current study are listed in Table 2. The primers were designed using Primer3 (http://www.ncbi.nlm.nih.gov/tools/primer-blast/).

**Table 2:** qRT-PCR SYBR Green primer sequences used in this study.

**Gene symbol**	**Forward primer (5^**′**^-3^**′**^)**	**Reverse primer (5^**′**^-3^**′**^)**
MUC1	TGCCGCCGAAAGAACTACG	TGGGGTACTCGCTCATAGGAT
FOXA1	GCAATACTCGCCTTACGGCT	TACACACCTTGGTAGTACGCC
HBA2	CTGGACAAGTTCCTGGCTTC	TGCTGCCCACTCAGACTTTA
MYOC	AGTTCCTGCTTCCCGAATTT	CTCGCATCCACACACCATAC
HBB	TCTGTCCACTCCTGATGCTG	CACTGGTGGGGTGAATTCTT
HBA1	GGTCCCCACAGACTCAGAGA	AGTGCGGGAAGTAGGTCTTG
β-ACTIN	AGAGCTACGAGCTGCCTGAC	AGCACTGTGTTGGCGTACAG

### RNA-Seq Data Analysis

Pair end reads were aligned to the hg19 human reference genome in CLC Genomics Workbench-12 (QIAGEN, Germany). The abundance of the expression of transcripts was measured as the score of TPM (Transcripts Per Million) mapped reads in CLC Genomics Workbench 12. Abundance data were subsequently subjected to differential gene expression using 2.0-fold change and < 0.05 *p-*value cut-off in CLC Genomics Workbench 12.

### Gene Set Enrichment and Modeling of Gene Interactions Networks

Upregulated genes were imported into the Ingenuity Pathways Analysis (IPA) software (Ingenuity Systems; www.ingenuity.com/) and were subjected to functional annotations and regulatory network analysis using upstream regulator analysis (URA), downstream effects analysis (DEA), mechanistic networks (MN) and causal network analysis (CNA) prediction algorithms. IPA uses precise to predict functional regulatory networks from gene expression data and provides a significance score for each network according to the fit of the network to the set of focus genes in the database. The *p-*value is the negative log of P and represents the possibility that focus genes in the network being found together by chance (12, 13).

### Retrieval of the Cancer Genome Atlas (TCGA) Breast Cancer Expression Data

Differentially expressed genes from the TCGA breast cancer data set were retrieved from (http://gepia.cancer-pku.cn/detail.php?gene=&clicktag=expdiy) (14). The expression profile of selected genes from the TCGA breast cancer data set was retrieved from the StarBase V3.0 database (http://starbase.sysu.edu.cn/panGeneDiffExp.php) (15).

### Statistical Analysis

Statistical analyses and graphing were performed using Microsoft excel 2016 and GraphPad Prism 8.0 software (GraphPad, San Diego, CA, USA). The Benjamini–Hochberg False Discovery Rate (FDR) method was used for multiple testing corrections. For comparative qRT-PCR analysis, *p*-values ≤ 0.05 (two-tailed *t*-test) were considered significant. For IPA analyses, a Z score (2.0 ≤ Z ≥ 2.0) was considered significant.

## Results

### RNA-Seq Gene Expression Profiling in BC

**Figure 1:**
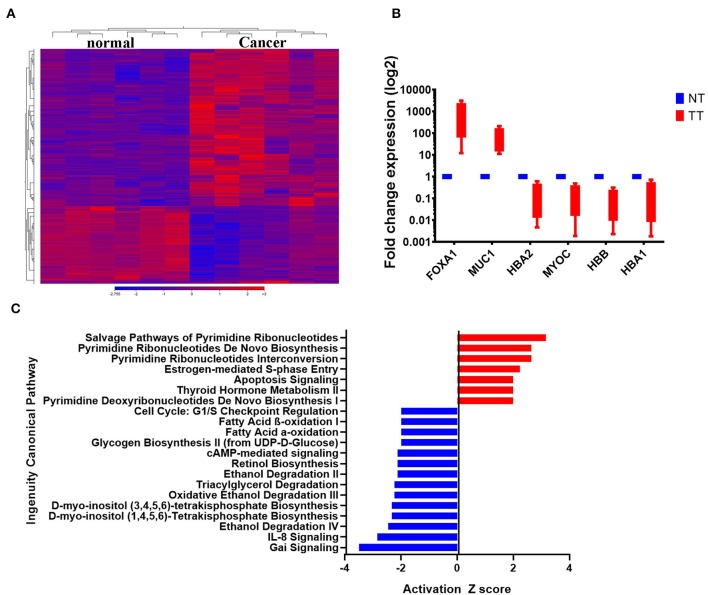
Differentially expressed genes in BCs. **(A)** Hierarchical clustering of six breast cancer and the corresponding adjacent normal tissue based on differentially expressed RNA transcripts (2.0 FC, FDR *p* < 0.05) from the RNA-Seq data. Each column represents a sample and each row represents a transcript. Expression level of each gene in a single sample is depicted according to the color scale. **(B)** The expression levels of selected genes from the RNA-Seq data were validated using qRT-PCR in four breast cancer and adjacent normal tissue. Data are presented as the mean ± S.E., *n* = 2. **(C)** Top significantly affected (2.0 <Z score< −2.0) canonical pathways based on IPA. The horizontal bars denote the different pathways based on the Z-scores. Red color indicate activation, while blue color indicate suppression.

The clinical information of the six patients included in the current study and their tumor characteristics are provided in Table 1. To characterize the transcriptional landscape alterations during malignant transformation, tumor, and adjacent normal breast tissues from six BC patients were subjected to whole transcriptome RNA-Seq analysis. As shown in Figure 1A, hierarchical clustering based on differentially expressed RNA transcripts revealed clear clustering of breast cancer from adjacent normal tissues. Using 2.0 FC and ≤ 0.05 FDR *p*-value cut off, 1108 upregulated and 518-downregulated transcripts were identified (Supplementary File 1). Selected number of upregulated (forkhead box A1 (FOXA1), mucin 1 (MUC1), and downregulated [hemoglobin alpha 2 (HBA2), myocilin (MYOC), hemoglobin subunit beta (HBB), and hemoglobin subunit alpha 1 (HBA1)] genes from the RNA-Seq data were subsequently validated using quantitative reverse transcriptase-PCR (qRT-PCR) (Figure 1B) and demonstrated concordant expression to those observed in the RNA-Seq data (Supplementary File 2). Canonical pathway analysis on the upregulated gene transcripts using ingenuity pathway analysis (IPA) revealed most significant enrichment in pathways related to pyrimidine ribonucleotides biosynthesis, and estrogen-mediated S phase entry, while G-Protein inhibitory (Gai) and IL8 signaling were among the most under-presented canonical pathways (Figure 1C).

### Activation of Cancer Cell Proliferation, Invasion, and Metabolism of DNA Functional Categories in Breast Cancer Tissue

**Figure 2:**
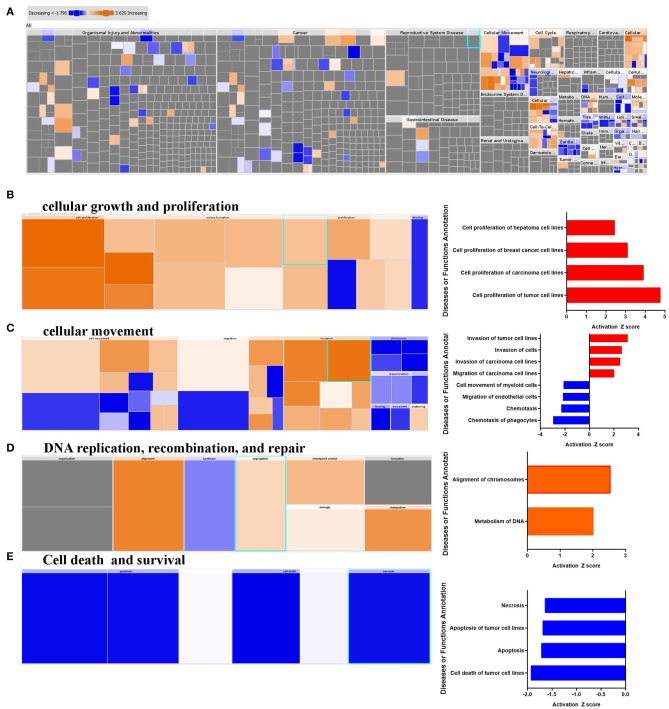
Downstream effector analysis of upregulated gene transcripts in breast cancer. **(A)** Tree map (hierarchical heat map) depicting affected functional categories based on differentially expressed genes where the major boxes represent a category of diseases and functions, **(B)** cellular growth, and proliferation, **(C)** cellular movements, DNA replication, recombination, and **(D)** repair, and **(E)** cell death and survival. Each individual colored rectangle is a particular biological function or disease and the color range indicates its predicted activation state: increasing (orange), or decreasing (blue). Darker colors indicate higher absolute Z-scores. In this default view, the size of the rectangles is correlated with increasing overlap significance.

IPA downstream effector analysis provides a powerful tool to predict the increase or decrease in downstream biological activities and functions that are likely to be casually affected by the transcriptome data. Figure 2A presents a high-level tree map of affected downstream functional categories based on differentially expressed genes in breast cancer tissue. The major colored rectangles indicates a family of associated biological functions or diseases, blue (decreasing) and orange (increasing), and dimension (using FET *P*-value) of rectangles indicates where associated functions are predicted to up or down most significantly as a group, the color intensity specify higher absolute Z-scores. This analysis revealed remarkable enrichment in several functional categories, mainly those involved in cancer cell growth, and proliferation (Figure 2B). Furthermore, functional categories associated with tumor cell movement and invasion were enriched, while those associated with myeloid and phagocyte cell chemotaxis were diminished (Figure 2C). Notably, functional categories associated with DNA replication, recombination and repair were also upregulated in BC tissues, especially those involved in chromosome alignment and metabolism of DNA (Figure 2D), while those involved in cell death were under presented (Figure 2E). Taken together, our data revealed a significant increase in cell proliferation, migration, DNA replication, while chemotaxis, and cell death-associated functional categories were suppressed.

### A Chemokine-Network Indicative of Enhanced Tumorigenesis in BC

**Figure 3:**
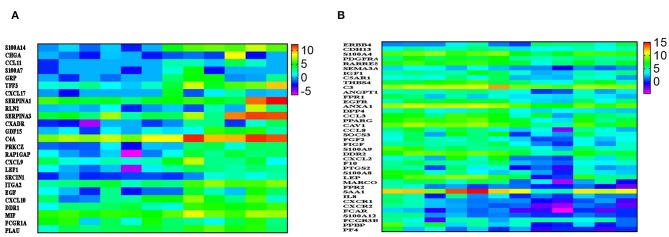
A chemokine-network indicative of enhanced tumorigenesis in BC. **(A)** Heatmap depicting the expression of several upregulated and **(B)** downregulated immune regulators in six breast cancer compared to adjacent normal tissue. Data are presented as log2 TPM expression value. Expression values are depicted according to the color scale.

IPA revealed a number of immune-related functional categories to be differentially expressed in BC compared to normal tissue. Among those, binding and movement of myeloid cells, and chemotaxis were most prominent (Figure 2C). Figure 3A, provides heat map log2 expression value for the upregulated immune-related genes (S100A14, CHGA, CCL11, S100A7, GRP, TFF3, CXCL17, SERPINA1, RLN2, SERPINA3, CXADR, GDF15, C4A, PRKCZ, RAP1GAP, CXCL9, LEF1, SRCIN1, ITGA2, EGF, CXCL10, DDR1, MIF, FCGR1A, and PLAU) in BC tissue. Interestingly, CXCL9, and CXL10 were shown before to enhance the mobilization of cytotoxic T cells form regional lymph nodes to tumor tissues and to promote CTL-mediated tumor immunity (16). In contrary, expression of CXCL17 by tumor cells was shown to recruit CD11b^+^Gr1^high^F4/80^−^ immune cells and to promote tumor progression in mice (17). On the other hand, several genes involved in chemotaxis were downregulated in breast compared to normal tissue (ANXA1, DPP4, CCL3, PPARG, CAV1, CCL8, SOCS3, FGF2, FIGF, S100A9, DDR2, CXCL2, F10, PTGS2, S100A8, LEP, MARCO, FPR2, SAA1, IL8, CXCR1, CXCR2, FCAR, S100A12, FCGR3B, PPBP, and PF4, Figure 3B). Previous studies had shown strong correlation between CCL2 expression and TAM infiltration and tumor progression (18). Interestingly, both CCL3 and CCL8 binds to CCR5, which was shown to regulate breast cancer cell proliferation, through P53 activation (19). Our data implies loss of CCL3 and CCL8 in breast cancer could lead to enhanced cell proliferation and tumor progression.

### Mechanistic Network Analysis Predicts Activation of ERBB2, FOXM1, ESR1, and IGFBP2 Networks in Breast Cancer

Upstream regulator analysis predicts upstream molecules and provides mechanistic networks that could explain the observed changes in gene expression. Upstream regulator analysis on the differentially expressed genes revealed several activated mechanistic networks in breast cancer, including ERBB2 (Z score = 4.5), FOXM1 (Z score = 3.9), FOXA1 (Z score = 2.5), ESR1 (Z score = 2.4), and IGFBP2 (Z score = 2.2), while suppression of NURP1 (Z score = −6.1), TP53 (Z score = −3.4) was prominent (Supplementary File 3).

**Figure 4:**
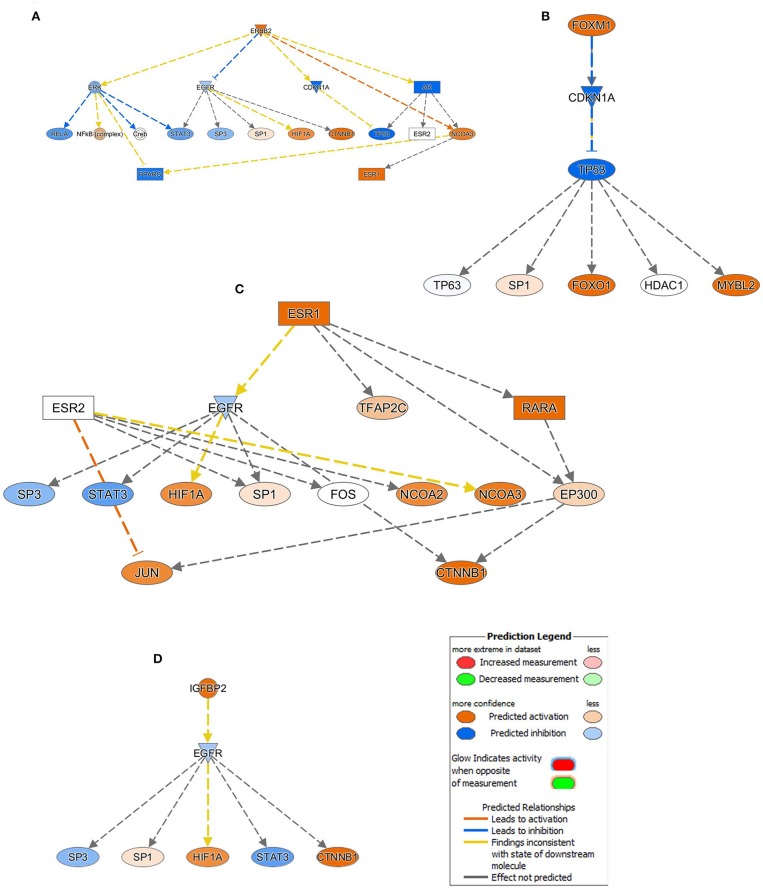
Mechanistic network analysis predicts multiple affected signaling networks. **(A)** Illustration of the ERBB2, **(B)** FOXM1, **(C)** ESR1, and **(D)** IGFBP2 mechanistic networks with predicted activated state of the network based on transcriptome data with subsequent predicted effects on downstream effector molecules. Figure legend illustrate the relationship between molecules within the network.

ERBB2 (HER2), which was upregulated in BC tissues, is predicted to be directly activating NCOA3 and inhibiting CDKN1A and AR (inconsistent relationship) with more confidence. Similarly, activated ERBB2 is directly inhibiting EGFR (inconsistent relationship) and ERK with less confidence, and in a high confidence state PPARG (inconsistent relationship) has been found to be inactivated through the down regulation of ERK and activation of NCOA3. Furthermore, upregulation of ERBB2 was predicted to activate the NFkB complex and impeding the RELA and STAT3 through the downregulation of ERK. However, its inconsistent relationship, in a high confidence mode tumor suppressor TP53 function, was disabled through direct inhibition of CDKN1A and AR. Moreover, HIF1A, CTNNB1, and ESR1 are predicted to be activated by the upregulated ERBB2 through the intermediated EGFR and AR downregulations (Figure 4A). Analogous to the ERBB2, FOXM1 also exert its inhibitory effect on TP53 via downregulation of CDKN1A (Figure 4B). While excavating further on the ESR1 function from the Figure 4A, though the effect of relationship was not predicted, RARA, NCOA2, NCOA3, HIF1A, JUN, and CTNNB1 are predicted in an active state whereas EGFR, SP3, and STAT3 are inactivated (Figure 4C). Similar occurrence has also been observed with IGFBP2 activation in BC tissues (Figure 4D).

### Breast Cancer Gene Signature Is Highly Enriched in Genes Indicative of Breast Cancer-Related Functional Categories With More Confidence and High Level of Predicted Relationship

**Figure 5:**
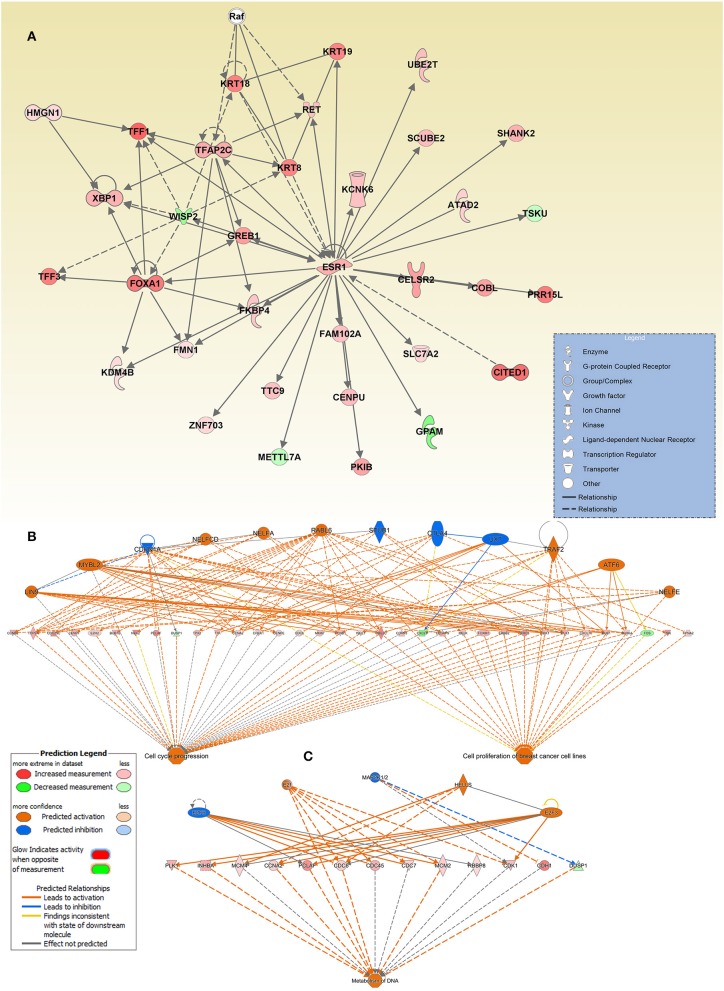
Enrichment in multiple cancer-associated networks in breast cancer. **(A)** Illustration of the “Cellular Development, Cellular Growth, and Proliferation, Digestive System Development and Function” network, “Cell cycle progression, **(B)** Cell proliferation of breast cancer cell lines”, and **(C)** “metabolism of DNA” functional networks based on IPA predicted activation state and subsequent effect on cellular functionality. Figure legend illustrate the relationship between molecules within the network and their activation state. Red color indicates activation while blue color indicate suppression.

Interestingly, we also observed ESR1 as a key hub gene in the cancer network. This interaction network is illustrated as genes (presented as nodes) and biological relationships between nodes (presented as edges) as mapped by IPA. The intensity of the node color correlates with the degree of gene upregulation. Nodes are displayed using different shapes representing the functional class of the gene (e.g., Enzyme, growth factors, transporters, etc.) that is illustrated in the corresponding legend (Figure 5A).

The top enriched functional network generated by the regulator effect network analysis in IPA is the cell cycle progression, and cell proliferation of breast cancer cell lines network. The network combined differentially expressed potential regulators (12) and genes (34, including 31 increased and 3 decreased legends) in the middle of the hierarchy which are involved in the two major downstream effector function such as cell cycle progression and cell proliferation of breast cancer cell lines (Figure 5B).

The other intriguing enriched network was that involved in the metabolism of DNA. This functional network consists of 3 activated (E2F, E2F3, and HELLS) and two inhibited (E2F6 and MAP2) upstream regulators, and 12 upregulated and one downregulated gene, in the middle hierarchy which are involved in metabolism of DNA (Figure 5C). Orange symbols at the top are the predicted upstream regulators. Colored symbols represent upregulated genes, with color intensity corresponding to the change in gene expression (Figure 5C).

**Figure 6:**
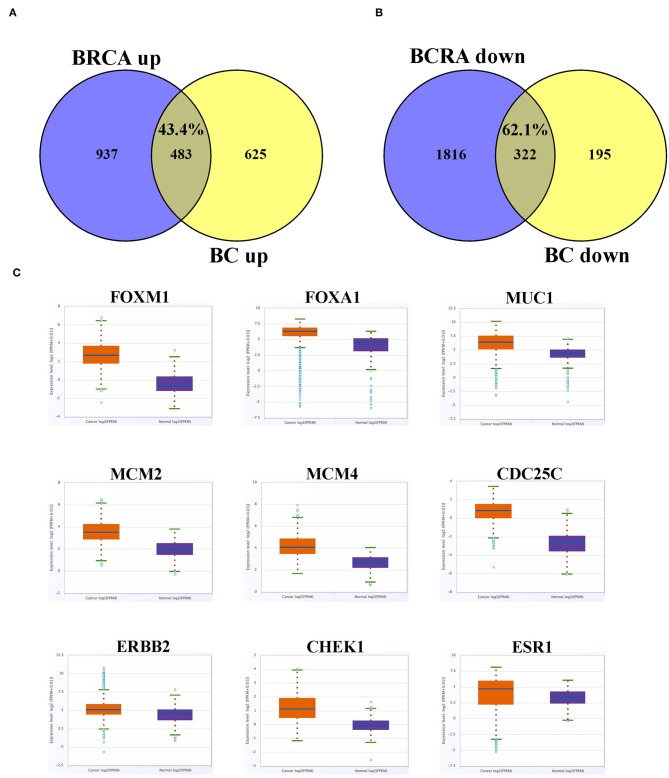
Overlap between differentially expressed gene in current study and the TCGA BC dataset. **(A)** Venn diagram depicting percentage overall in upregulated and **(B)** downregulated genes from current study compared to the TCGA BC dataset. **(C)** Expression of selected upregulated cancer-related genes based on current study in the TCGA BC dataset comparing BC and adjacent normal tissue. Red color indicate expression in breast cancer, while blue color indicate expression in adjacent normal tissue. Y-axis indicate expression intensity (log 2).

We subsequently compared the list of differentially expressed genes from the current study to those reported in the TCGA invasive breast cancer dataset. Overall, we observed a large similarity between the upregulated (43.5%) and downregulated (62.1%) genes in the current study and those reported in the TCGA invasive breast cancer dataset (Figures 6A,B). The expression profile of select cancer-related genes, which were upregulated in current study, in the TCGA dataset is presented in Figure 6C, which was concordant with our data.

## Discussion

The pathobiology of breast cancer is orchestrated by complex regulatory networks involving many gene hubs and regulatory molecules (20–22). Deciphering such complex signaling and functional networks provides a foundation for future development of targeted therapeutic interventions and disease biomarker discovery. While a multitude of transcriptomic data are currently available for breast cancer from several parts of the world, there are almost no such studies performed on breast cancer from the gulf region (23–25). Our previous data have highlighted a number of common and novel transcriptome networks in colorectal cancer from patients in the gulf region, suggesting a possible role for environmental and genetic factors in shaping the transcriptome of colorectal cancer (26, 27). Therefore, our current study provides the first RNA-Seq transcriptome analysis of breast cancer from the gulf region. Herein, we integrated the power of next generation sequencing with the ingenuity pathway analysis platform to understand the biology of breast cancer and to highlight various signaling and functional perturbations during breast cancer development in this geographic region, highlighting a number of key signaling networks in breast cancer.

Our global analyses revealed the enrichment of gene signatures indicative of cell proliferation and movement (migration and invasion), DNA replication and recombination, and immune cell trafficking, while genes associated with cell death were underrepresented. Mechanistic network analyses revealed activation of several signaling cascades with ERBB2, FOXM1, and ESR1 on top of the hierarchy. Our data are concordant with other studies highlighting an important role for ERBB (HER2), FOXM1, and ESR1 in breast cancer from other geographic regions (28–30).

In agreement with our expression data, functional studies revealed exogenous expression of ERBB2 in ERBB2-negative breast cancer cells (MCF7 and T47D) to enhance the expression of FOXM1 and MMP2. Inhibition of FOXM1 by RNA interference prevented induction of invasion by ionizing radiation (IR), while overexpression of FOXM1 in MCF10A cells was sufficient to promote IR-induced invasion (31). On the other hand, silencing of IGFBP-2 suppressed MCF7 breast cancer cell proliferation and increased cell death, suggesting IGFBP-2 as promoter of breast cancer survival (32). Interestingly, silencing of ER-α/ESR1 reversed the ability of IGFBP-2 to confer cell survival, suggesting IGFBP-2 to modulate IGFs, to directly regulate PTEN, and to play a role in maintaining ER-α expression (33). Those data corroborate a functional role for the identified molecular networks in BC biology.

Interestingly, one of the patients used in the RNA-Seq experiments was classified as HER2^−^ based on the pathological report; however, the expression of HER2 mRNA transcript was elevated based on the RNA-Seq data, suggesting possible differences in the pathological and molecular assessment of HER2 expression in breast cancer. Our data also revealed upregulation of not only ERBB2, but also ERBB3 and ERBB4 in breast cancer.

Tumor-infiltrating immune cells play critical roles in breast cancer pathogenesis (34). Our data highlighted the presence of a gene signature indicative of altered immune infiltration. Interestingly, CXCL9 and CXL10 were upregulated in BC tissue and were shown before to enhance the mobilization of cytotoxic T cells form regional lymph nodes to tumor tissues and to promote CTL-mediated tumor immunity (16). In contrary, expression of CXCL17 by tumor cells was shown to recruit CD11b^+^Gr1^high^F4/80^−^ immune cells and to promote tumor progression in mice (17). On the other hand, several other chemokines were downregulated in breast compared to normal tissue. For instance, previous studies had shown strong correlation between CCL2, which is downregulated in BC tissue, expression and TAM infiltration and tumor progression (18). Interestingly, both CCL3 and CCL8 binds to CCR5, which was shown to regulate breast cancer cell proliferation, through P53 activation (19). Our data implies loss of CCL3 and CCL8 in breast cancer could lead to enhanced cell proliferation and progression. Altered chemokine expression in the tumor microenvironment (TME) results into several consequences including leukocyte activation and trafficking, angiogenesis, metastasis, and proliferation of cancer cells (35, 36). In ovarian cancer, it has been reported that monoclonal antibodies or pharmacological inhibitors targeting CCL11 may be beneficial for the treatment of the disease (37). Additionally, both *in vivo* and *in vitro* studies in breast cancer patients elucidated the importance of CXCL17-CXCR8 axis in promoting the proliferation and migration of cancer cells (38). Additional studies on primary colorectal tumor showed that the expression of CXCL17 on tumor cells promotes angiogenesis and tumor infiltration of immune cells (39). These data show that CXCL17 could be a promising target for cancer immunotherapy. On the other hand, other reports showed that chemokine ligands including CXCL9 and CXCL10 have potential angiostatic and anti-tumor activities (40, 41). The interaction between CXCL9/CXCL10 with CXCR3 can recruit anti-tumoral dendritic cells, T lymphocytes and natural killer cells to the TME, which could be beneficial for tumor suppression (41). Our data suggest that in the breast TME, chemokines/receptors including CCL11, CXCL17, CXCL9, and CXCL10 were significantly upregulated, compared with normal tissues. Apart from chemokines, ITGA2 and EGF were also found to be upregulated (Figure 3A). It has been reported that ITGA2 is expressed more significantly in glioblastoma compared with normal glial cells, and targeting ITGA2 through monoclonal antibody could impede the migration of glioblastoma cells, but not their proliferation (42). Moreover, some reports showed that EGF promotes epithelial-mesenchymal transition (EMT), which could contribute to the migration/metastasis of tumor cells, and resistance to chemotherapy or hormonal therapy (43, 44). We also found that SERPINA1 and SERPINA3 to be upregulated in breast TME (Figure 3A). Previous reports showed that SERPINA1 and SERPINA3 are potential prognostic markers and therapeutic targets for colorectal cancer and melanoma, respectively (45, 46). Furthermore, another chemokine, CXCL17, which was found to be upregulated in breast tissue (Figure 3A), has been reported to be involved in angiogenesis, recruitment of immune suppressor cells and tumor metastasis (17, 47). CXCL17 was preferentially expressed in the aggressive types of breast, lung and gastrointestinal cancer cells, resulted in the accumulation of immature CD11b^+^Gr1^+^ myeloid-derived suppressor cells at tumor sites (17, 47). These data revealed that targeting migration-related genes including chemokines and their receptors in breast TME might be beneficial for tumor immunotherapy.

Current data revealed large similarity in the transcriptome of breast cancer from our study (43.5% upregulated), and (62.1% downregulated) when compared to differentially expressed genes from the TCGA BC dataset. In particular, we observed common altered breast cancer-driver genes in both datasets, suggesting common altered mechanism in breast cancer, regardless of the geographic region. Therefore, our data highlight a common molecular signature associated with key signaling networks in breast cancer, regardless of the ethnic background and geographical relation, which warrants further investigations using larger sample size and multicenter involvement.

## Data Availability

This manuscript contains previously unpublished data. The name of the repository and accession number(s) are not available.

## Ethics Statement

The study was performed under ethical approval from Qatar Biomedical Research Institute, Doha, Qatar (Protocol no. 2017-006). The patients/participants provided their written informed consent to participate in this study.

## Author Contributions

RV and VS wrote the manuscript. RV, VS, and KO performed the experiments. EE conceived study, obtained funds, revised and finalized the manuscript. NA analyzed data, revised and finalized the manuscript.

### Conflict of Interest Statement

The authors declare that the research was conducted in the absence of any commercial or financial relationships that could be construed as a potential conflict of interest.

## Abbreviations

BC: breast cancer
NGS: next generation sequencing
TT: tumor tissues
NT: normal tissues
IPA: ingenuity pathways analysis
URA: upstream regulator analysis
DEA: downstream effects analysis
MN: mechanistic networks
CAN: causal network analysis
TCGA: the cancer genome atlas
FOXA1: forkhead box A1
MUC1: mucin 1
HBA2: hemoglobin alpha 2
MYOC: myocilin
HBB: hemoglobin subunit beta
HBA1: hemoglobin subunit alpha 1
qRT-PCR: quantitative reverse transcriptase-Polymerase chain reaction.
