# Supplementary material for: Integrated Transcriptome and Pathway Analyses Revealed Multiple Activated Pathways in Breast Cancer
Source: Front Oncol. 2019 Sep 18;9:910. doi: 10.3389/fonc.2019.00910 (PMC6759650; doi:10.3389/fonc.2019.00910)
Supplement: Supplementary file 2 [file Table_2.DOCX]

**Supplementary file 2.** Fold change mRNA expression based on RNASEQ data analysis comparing six breast cancer to adjacent normal tissue (n=6). Two-tailed t test was used to compare the two groups.
